# Drugs of abuse and the adolescent athlete

**DOI:** 10.1186/1824-7288-36-19

**Published:** 2010-02-18

**Authors:** Alan D Rogol

**Affiliations:** 1 Riley Hospital for Children, Indiana University School of Medicine, Indianapolis, IN, USA; 2 University of Virginia, Charlottesville, VA, USA

## Abstract

Doping with endocrine drugs is quite prevalent in amateur and professional athletes. The World Anti-Doping Agency (WADA) has a list of banned drugs for athletes who compete and a strategy to detect such drugs. Some are relatively easy, anabolic steroids and erythropoietin, and others more difficult, human growth hormone (rhGH) and insulin like growth factor I (IGF-I). The use of such compounds is likely less in adolescent athletes, but the detection that much more difficult given that the baseline secretion of the endogenous hormone is shifting during pubertal development with the greatest rise in testosterone in boys occuring about the time of peak height velocity and maximal secretion of hGH and IGF-I.

This review notes the rationale, physiology, performance enhancement, adverse events and the detection of doping with insulin, rhGH, rhIGF-I, erythropoietin, and anabolic-androgenic steroids.

## Introduction

Can one grow more rapidly during childhood and early adolescence to a taller than genetically programmed adult height or to a more robust body composition given anabolic-androgenic steroids (AAS), human growth hormone (rhGH), insulin-like growth factor (rhIGF-I), insulin or erythropoietin? Does such growth permit an adolescent to increase his/her performance goals-- whether faster (citius) in many events, higher (altius) in jumping events and stronger (fortius)? Why would one consider that possible? The more successful late childhood and early adolescent age athletes are often more physically mature than their age-peers [1]. Early adolescent development seemingly permits them to use the strength and power in sport and performance that their average (or even slowly) developing age-peers have not yet attained. The exceptions might be those athletes in the more aesthetic sports in which flexibility and a lower center of gravity are more important than size and strength. These are virtually exclusively in females [1].

Most boys' sports require high levels of strength and power; however, at elite levels of competition technique is a strong measure of success; for the athlete must produce, control and efficiently use the energy in a fashion that maximizes athletic performance; for example, explosive power in some jumping sports or overall technical skill in the pole vault. Earlier developing children are taller and stronger than their age-peers. That may confer an advantage at younger ages; however, sport-specific skills are important. It is because of these that some of the "later blooming" adolescents catch-up in performance with their earlier developing peers and likely overtake them; for they have had the discipline to attain the requisite skills and are perhaps at lesser risk to "burn-out" and cease participation in that sport.

## Background

The rationale for taking ergogenic "effectors", such as rhGH, rhIGF-I, anabolic steroids, insulin or erythropoietin, is that by becoming bigger and stronger the athlete will perform better. Some boys who are not athletes take anabolic steroids and perhaps other ergogenic aides to "look better". However, there are no definitive data for rhGH or insulin in young adults and none at all for any of these five agents in adolescents who are not deficient in them. In any experimental setting the study design is such that one studies the effects of a single agent with **all other things being equal**. For a drug one decides on a dose, or range of doses and the subjects are allocated randomly to receive or not receive a particular dose. The subjects should be selected from a common pool and all should have identical "requirements", for example, in studies of athletes, prescribed diet and exercise regimens. It is apparent that many athletes take a "cocktail" of drugs making it virtually impossible to denote any single agent as causing a specific outcome.

Epidemiological data are available for anabolic steroids in adolescent high school athletes (4-12%), but not specifically in high level athletes. For the others there simply are no epidemiological data or data for doses, duration, pharmacokinetics or pharmacodynamics. It is quite unlikely that an ethical trial could be done in adolescents. It should also be noted that just as in adults, it is likely that adolescents combine dietary supplements with one or more illegal agents.

## Doping with Performance-Enhancing Endocrine Drugs

### Definition of Doping

The International Olympic Committee's (IOC) definition of doping is the *"use of an expedient (substance or method) which is potentially harmful to athletes' health and/or capable of enhancing their performance, or the presence in the athletes' body of a prohibited substance or evidence of the use thereof or evidence of the use of a prohibited method"*. There is no mention of intent or of how the substance entered the body. If the substance is in the athlete's body, then (s)he is responsible. That is the basis of sanctions for testing positive for a prohibited substance. Sir Arthur Porritt, first chairman of the IOC Medical Commission, noted: *"To define doping is, if not impossible, at best extremely difficult, and yet everyone who takes part in competitive sport or who administers it knows exactly what it means. The definition lies not in words but in integrity of character"*.

Such agents are also known as "performance enhancing substances" (PES) or "performance enhancing drugs" (PED). The American Academy of Pediatrics defines these agents as: *"...any substance when taken in non-pharmacological doses specifically for the purposes of improving sport performance. A substance should be considered performance enhancing if it benefits sports performance by increasing strength, power, speed or endurance (ergogenic) or by altering body weight or body composition *[2].

The agents that will be discussed are all hormones, rhGH, rhIGF-I, insulin, anabolic steroids and erythropoietin.

### Insulin

#### Rationale

The primary source of carbohydrate during exercise derives from muscle glycogen stores. The greater the amount of glycogen stored, the longer one should be able to exercise. Since insulin is a potent agent for the uptake of glucose and the subsequent glycogen storage in muscle, then the use of insulin ought to permit longer exercise time. In addition, insulin leads to the accumulation of amino acids in muscle and theoretically additional substrate for protein synthesis. Insulin may very well add to the anabolic properties of anabolic steroids and/or rhGH or rhIGF-I. The most likely scenario for a strength athlete would be to ingest a high carbohydrate diet, especially around the time of injecting rapidly acting insulin or its analogues.

#### Physiology

Insulin stimulates the uptake of glucose into muscle and fat by making available an increased number of glucose transporters (Glut-4) at the cell membrane thus increasing the flux of glucose to the interior of the cell. However, its main effect is inhibitory to lipolysis, glycolysis, gluconeogenesis, ketogenesis and proteolysis [3,4].

#### Performance enhancement

The theoretical efficacy to improve performance--muscle glycogen storage and the inhibition of proteolysis have not been shown in experimental protocols. That does not deter athletes from injecting insulin and its analogues, likely because insulin is but one of a "cocktail" or drugs along with training to enhance anabolic activity. In addition one must consider more rapid recovery from training and competition, but again there are no data to show this effect.

#### Adverse events

The main adverse event is the most obvious, that of hypoglycemia. Most athletes who abuse insulin are likely adept at balancing the ingestion of carbohydrate when injecting rapidly-acting insulin analogues. Much of the use of insulin, which is readily available, is likely covert, kept even from friends and family. It is unlikely that one would tumble to the diagnosis of hypoglycemia in young healthy, non-diabetic individuals when they present to medical evaluation with confusion or coma. That can be a fatal mistake.

#### Detection

There are no foolproof mechanisms for detecting insulin, unless one uses an analogue and that or one of its metabolites can be detected [5]. One would think that anti-insulin antibody detection techniques would be definitive, but there is a low frequency of circulating anti-insulin antibodies in some normal (non-diabetic) individuals.

### Human Growth Hormone

#### Rationale

The rationale for adolescent athletes taking rhGH, is that it should synergize with the physiologic (or doping) increases in testosterone to grow faster and to add additional lean body mass [6]. It is understood that by becoming bigger and stronger the athlete will perform at a higher level. hGH is lipolytic in addition to being anabolic and thus may be considered for those in "physique" related sports such as body-building. In multiple surveys of adolescents who take anabolic agents, it is to "look good" as often as it is to augment athletic performance [7-9]. As noted previously athletic performance is much more than just strength or endurance; for the athlete must produce, control and efficiently use the energy in a fashion that maximizes sport performance.

#### Physiology

The most notable effect in children and adolescents is linear growth. Growth hormone-deficient children and adolescents are likely to be 20 to 30 cm shorter than mid-parental target height if left untreated. Physiological stimuli to hGH release include: deep sleep, some amino acids and exercise. hGH is secreted in a pulsatile fashion every hour or two and has significant peaks approximately 90 min after the onset of deep sleep and within minutes of completing a bout of exercise. The main (iso)form of hGH is a 191 amino acid, 22 kD peptide with a significant amount of a 20 kD splice variant form. There are a multitude of post-translationally modified forms including those affected by metabolism and sulfation and phosphorylation. Several of the forms, predominantly the 22 kD and 20 kD forms interact with the hGH receptor, a dimeric member of the cytokine family of receptors at the cell surface to lead to the cellular actions of hGH. One of the main ones is to stimulate the hepatic synthesis of IGF-I (endocrine), but also to stimulate the paracrine and autocrine synthesis of IGF-I in various tissues, such as bone and muscle [10].

In addition, it is lipolytic and can significantly alter body composition by directing muscle protein synthesis relative to fat. In fact, it is this action that has led to its use in animal husbandry with the "partitioning" of feed and "feed-efficiency" as the major outcomes [10].

#### Performance enhancement

Despite the paucity of data for performance enhancement, the "word on the street" is that this agent is quite actively being abused by athletes. It is unlikely that the athlete uses rhGH in isolation, thus making it quite difficult to define its exact role in athletic performance. There is however one very well controlled study that shows an effect on strength in a group of well defined abstinent steroid abusers (subject to strict drugs of abuse testing) [11]. There are no studies in child and adolescent athletes, but it may be that faster growth, weight gain and the salutary effects on body composition during adolescence could confer advantage to age-group athletes, for it has been shown at the elite level that the taller, heavier early adolescent athlete is represented out of proportion to his/her age-peers [1].

Adolescents reach "near-adult" height later in the teen years with girls reaching this stature several years before boys; however, the adult body composition (muscle, bone and fat) and the regional distribution of body fat are not attained until early-to-middle third decade. If rhGH is effective to help one obtain these milestones early, perhaps there is some advantage; however this is quite theoretical [6].

#### Adverse events

There are many data for the **short-term **(years to more than a decade) in children with a number of conditions of childhood: GH deficiency, several genetic condition as well as "short-normal" children. Two major data bases, the Kabi International Growth Study (KIGS), now managed by Pfizer, Inc and the National Cooperative Growth Study produced and managed by Genentech, Inc show quite low incidences of scoliosis, slipped capital femoral epiphysis and rare instances of idiopathic intracranial hypertension, the one of greatest concern. Adults have noted more muscle and joint complaints than children. There is the long-term (theoretical) issue of neoplasia.

#### Detection

The World Anti-Doping Agency (WADA) has placed rhGH on its banned list and blood tests are available for its detection. These have in their present iteration a relatively small "window" for detection of doping. There are two major methods for detection, the isoform method [12] and the "markers" method [13], which have been well-described and the details are beyond the purpose of this review.

### Insulin-like growth factor I

#### Rationale

The rationale for using rhIGF-I as an ergogenic aide differs little from that of rhGH. This compound has only recently become more available and its use in athletes will only increase. The potential benefits include increased muscle protein synthesis and the sparing of glycogenolysis with glycogen synthesis and increased fatty acid availability

#### Physiology

IGF-I is the main effector for the action of hGH--see above. Whereas hGH is insulin antagonistic several hours after ingesting a meal, the main effect of hIGF-I is to reduce glucose levels (insulin-like) and it is this effect that has been noted clinically (see below). It is strongly anabolic in muscle, but has a very much diminished effect in comparison to hGH on lipids. In fact, children with virtually no IGF-I (growth hormone receptor deficiency, Laron-type) gain a disproportionate amount of fat when treated for many years with rhIGF-I [14].

#### Performance enhancement

This is unknown at present. There just haven't been studies in adults or children. rhIGF-I has been used as a growth promoting agent in children with both primary IGF-I deficiency and in a few genetic condition which are associated with short stature. It is quite early for the "efficacy" results, but growth rates have increased and it is the only presently available drug for those with complete growth hormone insensitivity.

#### Adverse events

The major adverse event in children with growth hormone insensitivity has been hypoglycemia; however, most of the instances can be overcome with food ingestion around the time of the administration. Some unique side effects include jaw pain, headache, fluid retention and myalgia. As with rhGH there has been an incidence of idiopathic intracranial hypertension. Similar to the findings with rhGH stopping the drug for several days and perhaps restarting at one-half dose seems prudent in those who have experienced this adverse event.

In the longer term there is the theoretical aspect of tumorigenesis, although no data exist, other than associative ones for patients with certain cancers (for example, breast, prostate, and colon) have had higher IGF-I levels in the years before their cancers became detected [15].

#### Detection

There is no specific test for rhIGF-I to detect doping. The most likely approach will be to use the "markers" method, described above for growth hormone [16].

### Erythropoietin (EPO)

#### Rationale

Erythropoietin leads to the production of red blood cells. Since these carry oxygen to active muscles, one would consider enhanced endurance performance because of the additional flux of oxygen.

#### Physiology

EPO is a circulating glycosylated protein hormone that is the principal regulator of erythropoiesis. It is produced primarily by the kidney inversely related to the concentration of O_2 _in the blood. Following administration, there is a direct relationship between haemoglobin levels and increased performance following administration of rHuEPO in rats and humans [17]. Methods used in doping include hypoxia and hypoxia-mimetics. One such method is to train at altitude; however, one cannot necessarily train as hard as at lower altitude because of hypoxic-mediated fatigue. Variations on this theme include living at altitude and training at sea level or sleeping in a tent or chamber with diminished oxygen tension (at lower altitude) and training at the same elevation. These methods are not considered doping.

#### Performance enhancement

rHuEPO and its follow-on biological relatives can provide an effective mechanism to stimulate erythropoiesis as noted above; however, the baseline hematocrit increases and may rise even more to dangerous levels, likely due to dehydration, in athletes during and after training and competition. The rheology of blood changes exponentially as the hematocrit rises above 55% and accelerates even more rapidly as it rise above 60%.

#### Adverse events

Deaths in competitive cyclists have been directly linked to changes in the flow properties of blood, as the hematocrit rises. There are no studies of rHuEPO, or its related proteins in child or adolescent athletes, but theoretically the responses should be no different from older adolescents or young adults. The major issues are those that relate to increased hematocrit- sluggish blood flow in the small vessels of critical organs and pulmonary emboli.

#### Detection

Detection of EPO and some of the follow-on drugs is performed on urine sample using gel electrophoresis. Each of the individual compounds (drugs) has a "signature" of glycosylation sites making the detection quite precise [18].

### Anabolic steroids

#### Rationale

Anabolic steroids regulate the building blocks for the adolescent growth spurt and body composition. Even the body uses the combination of testosterone, hGH and IGF-I to subserve the adolescent growth spurt and the "partitioning" (see above) of food energy into the various compartments of body composition. As noted previously the early attainment of pubertal development confers some advantage on adolescent athletes compared to their "on-time" peers, although this is likely less important later in puberty given that sport-specific skills become more important as the majority of athletes will have entered and progressed through pubertal development.

#### Physiology

An anabolic steroid is a sex hormone that promotes the development and maintenance of the male sex characteristics. Testosterone is the principal secreted androgen in men. Androgens have both masculinizing (development of male secondary sex characteristics, including hair growth) and anabolic effects (increase in skeletal muscle mass and strength). For decades, pharmaceutical companies have attempted to develop androgens that have preferential anabolic activity and reduced or no androgenic activity; these compounds have been referred to as anabolic steroids.

There is feedback control of androgen synthesis and secretion involving the hypothalamus (GnRH) and the pituitary. During adolescence there are remarkable alterations in both secretion and feedback sensitivity that underpin male pubertal development. Testosterone is strongly bound to sex-hormone binding globulin (SHBG), loosely bound to albumin, and a small proportion circulates as the free hormone. The biological activity is thought to reside in the free and loosely bound (albumin-bound) fractions [19].

Testosterone causes the hypertrophy of muscle fibers [20] and an increase in the number of myonuclei and satellite cells [21]. This metabolic action follows from the binding of testosterone to the androgen receptor. Its ability to transduce the binding of testosterone in the cytoplasm of the cell to its anabolic action (altering gene function in the nucleus) is inversely proportional to the number of CAG repeats in the first exon of the androgen receptor, a member of the nuclear transcription factor family of receptors and located in virtually all tissues [19].

#### Performance enhancement

There are many reports of muscle hypertrophy and increases in strength in athletes who take anabolic steroids. Virtually none are controlled trials of just testosterone or one of other anabolically-active steroids, for many ingest and inject multiple potentially active drugs. This subject has recently been exhaustively reviewed [19].

The difficulty in adolescents is that increased secretion of testosterone (and growth hormone) is a natural part of human pubertal development. Thus it would be very difficult to ascribe any individual changes noted in strength and body composition to any pharmacological agent at this time. The ethics of human experimentation in adolescents precludes any serious objective study using anabolic steroids

#### 3.5.4 Adverse events

Adverse events in adolescents include those noted in the adult--acne, shutting down of the hypothalamic-pituitary-gonadal axis, unfavorable lipid profiles, hepatic dysfunction, psychological disturbances (so-called 'roid' rage) and other less common effects (reviewed extensively in [19]). However, in addition, since testosterone is a precursor to estrogens, there will be an acceleration of epiphyseal maturation of the long bones and shorter than predicted adult stature. In girls and women hirsutism and disordered ovarian and menstrual cycles can occur with quite small doses of anabolic steroids.

#### Detection

Much has been written in this area and I shall not review it here. Methods using liquid chromatography and mass spectrometry are the standard and "libraries" of multiple compounds and their fragmentation products help to define the precise steroid taken. As noted several years ago for tetrahydrogestinone, once the pure compound is available a test can be validated very quickly [22].

## Conclusion

It is likely that drugs of abuse in adult athletes are also used in adolescent athletes. The data for the later are mainly theoretical because proper studies cannot be ethically performed and it is unlikely that any of the agents are taken in isolation.

## Competing interests

The author declares that he has no competing interests.

## Authors' contributions

The author chose the subject and wrote the entire manuscript.
